# RNA editing regulates lncRNA splicing in human early embryo development

**DOI:** 10.1371/journal.pcbi.1009630

**Published:** 2021-12-01

**Authors:** Jiajun Qiu, Xiao Ma, Fanyi Zeng, Jingbin Yan

**Affiliations:** 1 Shanghai Children’s Hospital, Shanghai Institute of Medical Genetics, Shanghai Jiao Tong University, Shanghai, China; 2 NHC Key Laboratory of Medical Embryogenesis and Developmental Molecular Biology & Shanghai Key Laboratory of Embryo and Reproduction Engineering, Shanghai, China; 3 Group of Signal Transduction, Department of Psychiatry and Psychotherapy, University Hospital, LMU Munich, Munich, Germany; University of Missouri, UNITED STATES

## Abstract

RNA editing is a co- or post-transcriptional modification through which some cells can make discrete changes to specific nucleotide sequences within an RNA molecule after transcription. Previous studies found that RNA editing may be critically involved in cancer and aging. However, the function of RNA editing in human early embryo development is still unclear. In this study, through analyzing single cell RNA sequencing data, 36.7% RNA editing sites were found to have a have differential editing ratio among early embryo developmental stages, and there was a great reprogramming of RNA editing rates at the 8-cell stage, at which most of the differentially edited RNA editing sites (99.2%) had a decreased RNA editing rate. In addition, RNA editing was more likely to occur on RNA splicing sites during human early embryo development. Furthermore, long non-coding RNA (lncRNA) editing sites were found more likely to be on RNA splicing sites (odds ratio = 2.19, P = 1.37×10^−8^), while mRNA editing sites were less likely (odds ratio = 0.22, P = 8.38×10^−46^). Besides, we found that the RNA editing rate on lncRNA had a significantly higher correlation coefficient with the percentage spliced index (PSI) of lncRNA exons (R = 0.75, P = 4.90×10^−16^), which indicated that RNA editing may regulate lncRNA splicing during human early embryo development. Finally, functional analysis revealed that those RNA editing-regulated lncRNAs were enriched in signal transduction, the regulation of transcript expression, and the transmembrane transport of mitochondrial calcium ion. Overall, our study might provide a new insight into the mechanism of RNA editing on lncRNAs in human developmental biology and common birth defects.

## Author summary

RNA editing is a process by which selected nucleotides on RNA molecules are changed post transcription of RNA. This results in changes RNA sequences that are different from the corresponding DNA sequence at modified positions. However, this slight difference has a great impact on various biological processes. In this manuscript, we investigated the function of RNA editing during human early embryo development. Through analyzing the sequencing data and computational biology, we found that RNA editing could regulate the alternative splicing of long-noncoding RNAs (lncRNAs) in human early embryo development. Here, lncRNAs are special kind of RNAs that do not translate into proteins, but have functions in the regulation of other genes. Alternative splicing is a process through which RNA molecules can be spliced into different transcripts after transcription. Thus, our results indicated that RNA editing could regulate the alternative splicing of lncRNAs and further affect the expression of downstream genes in human early embryo development. RNA editing could be the reason for the tremendous change of gene expression profile in human early embryo development which was found the previous researches.

## Introduction

Early embryo development is a complicated biological process in which a large number of genes and factors are involved. Dynamic changes in gene expression were found during human early embryo development [1]. Each developmental stage can be delineated concisely by a small number of functional modules of co-expressed genes. The sequential order of transcriptional changes in pathways of the cell cycle, gene regulation, translation, and metabolism, act in a step-wise manner from cleavage to morula [2]. However, the molecular mechanism behind the dynamic changes in gene expression during early embryo development is still unclear. For example, zygotic genome activation (ZGA) at 8-cell stage promotes a remarkable reprogramming of gene expression patterns, coupled with the generation of novel transcripts that are not expressed in oocytes. However, the mechanism by which ZGA achieves such reprogramming needs to be further studied [3].

Long non-coding RNAs (lncRNAs), which are typically >200 nucleotides in length, are involved in human early embryo development [4]. LncRNAs have a stage-specific expression pattern during human early embryo development [5], and this special expression pattern is related to human oocyte maturation and human ZGA [5]. However, there are still many unknowns behind the association between lncRNA and human early embryo development. For example, the regulation leading to the stage-specific expression pattern of lncRNA remains unclear.

RNA editing is a molecular process perturbing RNA sequences in a co- or post-transcriptional manner. Thus far, >100 distinct types of RNA modifications have been identified [6]. In mammals such as *Homo sapiens*, the most prevalent form of RNA editing is the single nucleotide change of adenosine (A) to inosine (I) by double-stranded RNA-specific adenosine deaminase enzymes [7]. The effect of RNA editing can be diverse, depending on the location of the edited nucleotide. RNA editing on mRNA can create new start and stop codons by uridine insertion and cytidine to uridine (C-to-U) conversions, which consequently alters the protein-coding sequences of the selected genes, resulting in a diversification of their protein functions [8,9]. RNA editing on tRNA creates new essential structural elements by nucleotide deletion/insertion, nucleotide insertion, or base conversion. Some types of RNA editing on tRNA change its identity (i.e. alters its recognition by aminoacyl synthases), whereas others may affect 5’ and/or 3’ processing [9]. Thus far, RNA editing was only found to affect the expression of lncRNAs [10]. Despite the effects of RNA editing on lncRNA are still not well studied, a previous study found there is a regional- and cell type-specific regulation of RNA editing of a set of target transcripts, which indicates that RNA editing could have a stage-specific regulation on lncRNA transcripts during early embryo development [11].

This study focused on the functions of RNA editing on lncRNAs. Based on single cell sequencing data, it was found that RNA editing can regulate lncRNAs splicing during human early embryo development.

## Materials and methods

### Single cell sequencing data

The dataset GSE44183 from NCBI contained 29 samples including 3 oocyte samples, 2 zygote samples, 3 2-cell stage samples, 4 4-cell stage samples, 11 8-cell stage samples, and 3 morula stage samples, which were consisted of pair-end sequencing data based on the Illumina HiSeq 2000 platform (Illumina, Inc.) [2]. The dataset GSE101571 was also used to validate the results, which include two oocyte samples, three 2-cell stage samples, two 4-cell stage samples, and two 8-cell stage samples.

### Mapping of RNA-seq reads

The HISAT2 algorithm was used to align RNA-seq reads to the reference genome (hg19) with the parameter ‘—mp 6,3’ [12]. The Mark Duplicates tool from Picard (http://picard.sourceforge.net/) was used to remove identical reads (PCR duplicates) that mapped to the same location. The GATK tool BaseRecalibrator was used to obtain more accurate base qualities, which in turn improves the accuracy of the variant calls [13].

### Variant calling and filtering

The whole pipeline was available at https://github.com/JiajunQiu/RNAediting_Pipeline. The variants were first called by GATK HaplotypeCaller with the option ‘stand_call_conf’ set as 0. Variants were required to be supported by at least two mismatched reads with a base quality score ≥25 and a mapping quality score ≥20 [13].

All known SNPs present in dbSNP were removed (except SNPs of molecular type ‘cDNA’; database version 150; http://www.ncbi.nlm.nih.gov/SNP/). Variants detected in at least two individuals were kept because they were unlikely to be rare variants [13]. Possible false-positive RNA editing sites due to sequencing stand were removed by taking advantage of their tendency to biased positioning on sequencing reads and to biased proportions of sequencing strands. It meant that we removed the editing sites whose variant-supporting reads were significantly from only one strand. After categorizing sequencing reads spanning a putative RNA editing site into two groups, namely a reference-supporting group and a variant-supporting group, according to the alleles in the reads, a Fisher’s exact test was applied to analyze whether the two groups were statistically different in terms of position and strand, respectively [14]. For a certain site, if the P-value from the test was smaller than a threshold, the site was considered a false positive. In this study, P = 0.01 was considered the P-value threshold, and Bonferroni’s correction was used for multiple-group comparisons [14].

RNA editing candidates were removed if they were located in regions of high similarity to other parts of the genome. For that purpose, BLAT was applied to all reads that overlapped an RNA candidate site and at the same time showed a mismatch from the reference. For each read, it was required that i) the best hit overlapped the candidate site and ii) the second-best hit had a score <95% of the best BLAT hit. Only sites for which the number of reads passing the above BLAT criteria was larger than the number of reads that failed the criteria were kept [15].

Finally, reliable RNA editing sites were identified when the mismatch frequency was ≥0.1, and there were at least two individuals who had ≥5 high-quality (PHRED score ≥20) reads and ≥2 high-quality variant-supporting reads for an RNA editing site candidate. Sites with two or more variants were excluded [14].

Annotation of RNA editing site was conducted by ANNOVAR [16,17]. RNA annotattion was based on the following databases: UCSC [18], NONCODE [19], GENCODE [20], CHESS [21], LNCipedia [22], FANTOM [23], MiTranscriptome [24] and BIGTranscriptome [25]. Annotation of transcription factor binding site (TFBS) was performed with the tfbsConsSites database from UCSC (http://hgdownload.cse.ucsc.edu/goldenPath/hg19/database/tfbsConsSites.txt.gz) [26]. The SPIDEX annotation database was used for RNA splicing site annotation [27].

Two matched sets of DNA exome sequencing (exome-seq) and RNA-seq (GSE94813) were generated to evaluate the performance of the computational pipeline identifying RNA-editing sites under the assumption that observed DNA and RNA sequence differences were mainly caused by RNA editing [28]. The RNA editing sites identified through the above pipeline were considered true if the corresponding genomic sites had homozygous genotypes with the reference alleles; otherwise, they were considered false. Specifically, RNA editing sites were first identified from individual RNA-seq through the above pipeline, except for the step requiring multiple samples. Next, genotypes for RNA editing sites were called from matched exome-seq. Those RNA editing sites that were found in both DNA and RNA sequencing data were considered false positives [14]. The false discovery rates (FDRs) of the two matched sets were 0.48 and 0.59%, respectively. It should be noted that the additional filters in the pipeline, which took advantage of the multitude of samples, were not used for this evaluation, but would be expected to further decrease the FDR in our results.

Differentially edited editing sites across developmental stages were identified by ANOVA among the stage groups, followed by multiple test corrections with FDR. An FDR-adjusted P<0.05 was considered to indicate a statistically significant difference [14].

### Competing tools

We compared our RNA editing site identification pipeline with the following four tools for detecting RNA editing: GIREMI [29], REDItools [30], RNAEditor [31], and SPRINT [32]. According to previous study [32], U87MG dataset was used to compare our pipeline with other RNA editing detecting methods. The U87MG dataset includes the RNA-seq data of U87MG ADAR knockdown sample [33]. It can be used to assess the FDR of methods. By assuming that all RNA editing sites detected in U87MG ADAR knockdown sample are false positives, the FDR of a given method was calculated as the ratio of the number of A-to-I RESs detected from U87MG ADAR knockdown sample to the number of A-to-I RESs detected from U87MG ADAR control sample.

### Single-cell data analysis

The R package Seurat (https://satijalab.org/seurat/) was applied to normalize and analyze the single cell data. After that, the R package Monocle 3 was applied to order cells in pseudotime along a trajectory (https://cole-trapnell-lab.github.io/monocle3). After reducing the dimension with uniform manifold approximation and projection (UMAP) method, the cells were ordered according to their progress through the developmental program. Monocle estimates this progress in pseudotime.

### Identification of differentially expressed exons

Differentially expressed exons identification was performed with the HTseq-DEseq2 pipeline based on the annotation databases: UCSC [18], NONCODE [19], GENCODE [20], CHESS [21], LNCipedia [22], FANTOM [23], MiTranscriptome [24] and BIGTranscriptome [25]. Adjusted P<0.05 was used as the threshold for differential expression [34,35].

### Percentage spliced index (PSI)

To confirm the differential transcripts splicing, the PSI of each exon was calculated according to a previously developed protocol [36]. The PSI is the ratio between reads including or excluding exons. It is also known as ‘percent spliced in index’, and it indicates how efficiently the sequences of interest are spliced into transcripts.

### Correlation between RNA editing rate and exon expression or PSI

The RNA editing rate of each exon was normalized as the total number of Gs divided by the total number of Gs + As in each exon. Pearson’s correlation coefficient was calculated between the RNA editing rate and the expression level or PSI of each exon.

### GO annotation enrichment analysis of lncRNAs

GO annotations of lncRNAs were obtained from the published algorithm: lncRNA2GOA [37]. Enrichment analysis was performed with the R package topGO [38]. Treemap of GO enrichment result was drawn with R package rrvgo [39]. And the threshold was set to be: adjusted p value < 0.01.

## Results

### Single-Cell Trajectory Analysis reveals the pseudoearly embryo development timeline

**Fig 1:**
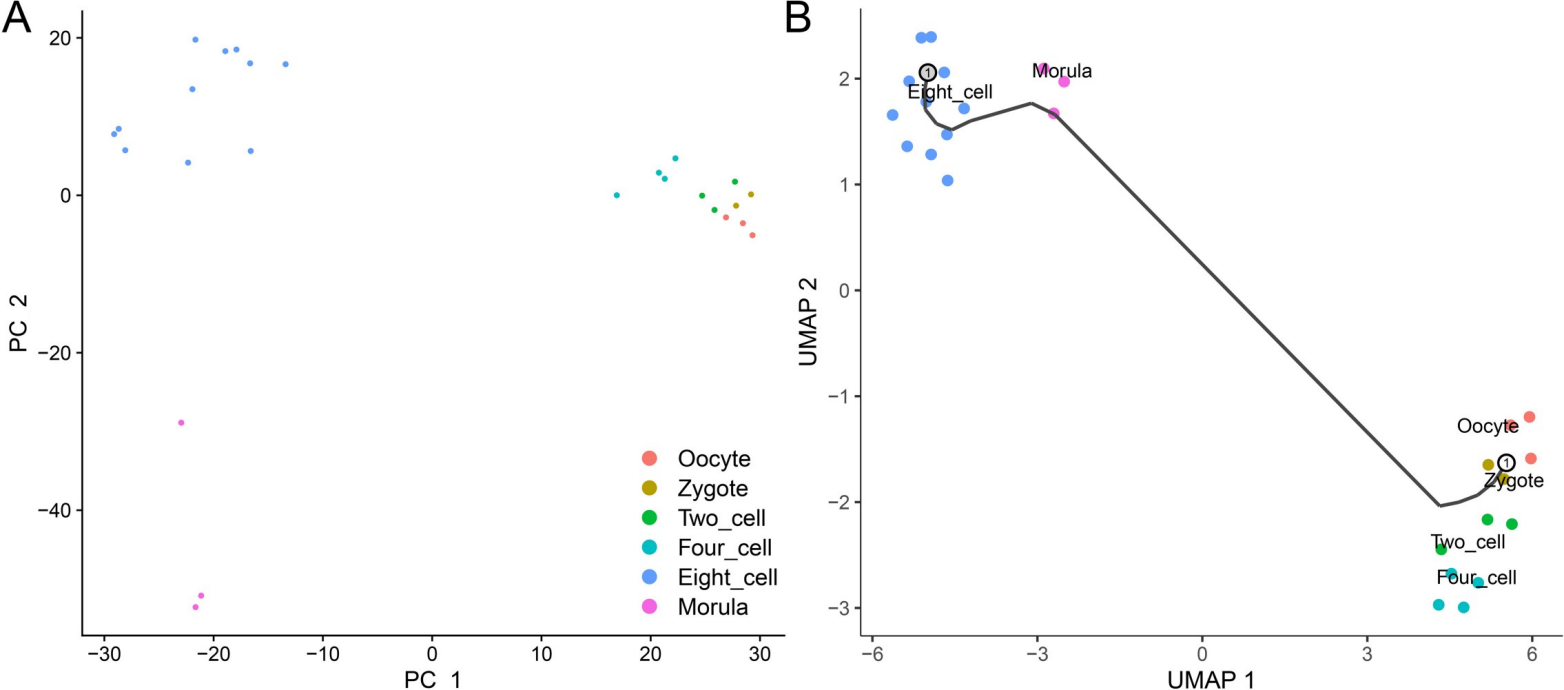
General expression analysis. (A) Principal Component Analysis (PCA). Samples are separated well with the first two principal components. (B) Single-cell trajectory analysis. Samples are visualized with uniform manifold approximation and projection (UMAP). The line is the pseudo timeline learned by Monocle 3, corresponding to the real human early embryo development.

First of all, as requested, we did some general expression analysis for the data. After normalizing and scaling the expression matrix, we performed the principal component analysis (PCA) on the data. The result showed that samples from different development stages were clearly separated (Fig 1A). Especially, the samples from the 8-cell stage and Morula stage were extremely isolated from the others. It indicated that there was a big change of gene expression pattern after the 8-cell stage, which makes the samples after the 8-cell stage different from the rest. Then, we used UMAP to reduce the dimension of the data and performed Single-Cell Trajectory Analysis with R package Monocle 3. We could see a clear pseudo early embryo development timeline on the dataset (Fig 1B). It just corresponded to real human early embryo development process ranging from oocyte to morula stage (Fig 1B). In this study, we performed different expression (DE) analysis with the classic method DEseq according to the previous publication [2]. But we also compared DEseq with other DE analysis methods. From the result, we can see a large overlap between the DEseq and DEsingle (S1A Fig). On the other hand, method MAST showed different results comparing with the former two (S1B and S1C Fig). The reason could be DEseq and DEsingle are better at the raw read count data, while MAST is more suitable for transcripts per million (TPM) data.

### RNA editing site detecting pipeline produces reliable identification of RNA editing sites

Then, we compared the performance of our RNA editing site identification pipeline with other published methods. Corresponding to the previous study [32], RNA editing sites were divided into three categories: Alu, repetitive non-Alu, and non-repetitive regions. For Alu RNA editing sites, GIREMI had the lowest FDR (0.70%) (S2 Fig), and our pipeline ranked second and achieved a comparable number of FDR = 1.99% (S2 Fig). And for repetitive non-Alu RNA editing sites, SPRINT had the lowest FDR 4.50% (S2 Fig), our pipeline ranked second again with an FDR = 8.68% (S2 Fig). Regarding nonrepetitive RNA editing sites, our pipeline still ranked second in the comparison after the method SPRINT (S2 Fig). It was hard to conclude which method was the best RNA editing site detector since none of the methods can achieve the highest performance in all three categories. However, as a method that ranked the second in the comparison of all three categories of RNA editing sites, at least, we could claim that our pipeline produced the high reliable identification of RNA editing sites for the following analysis (S2 Fig).

### RNA editing landscape across human early embryo development

**Fig 2:**
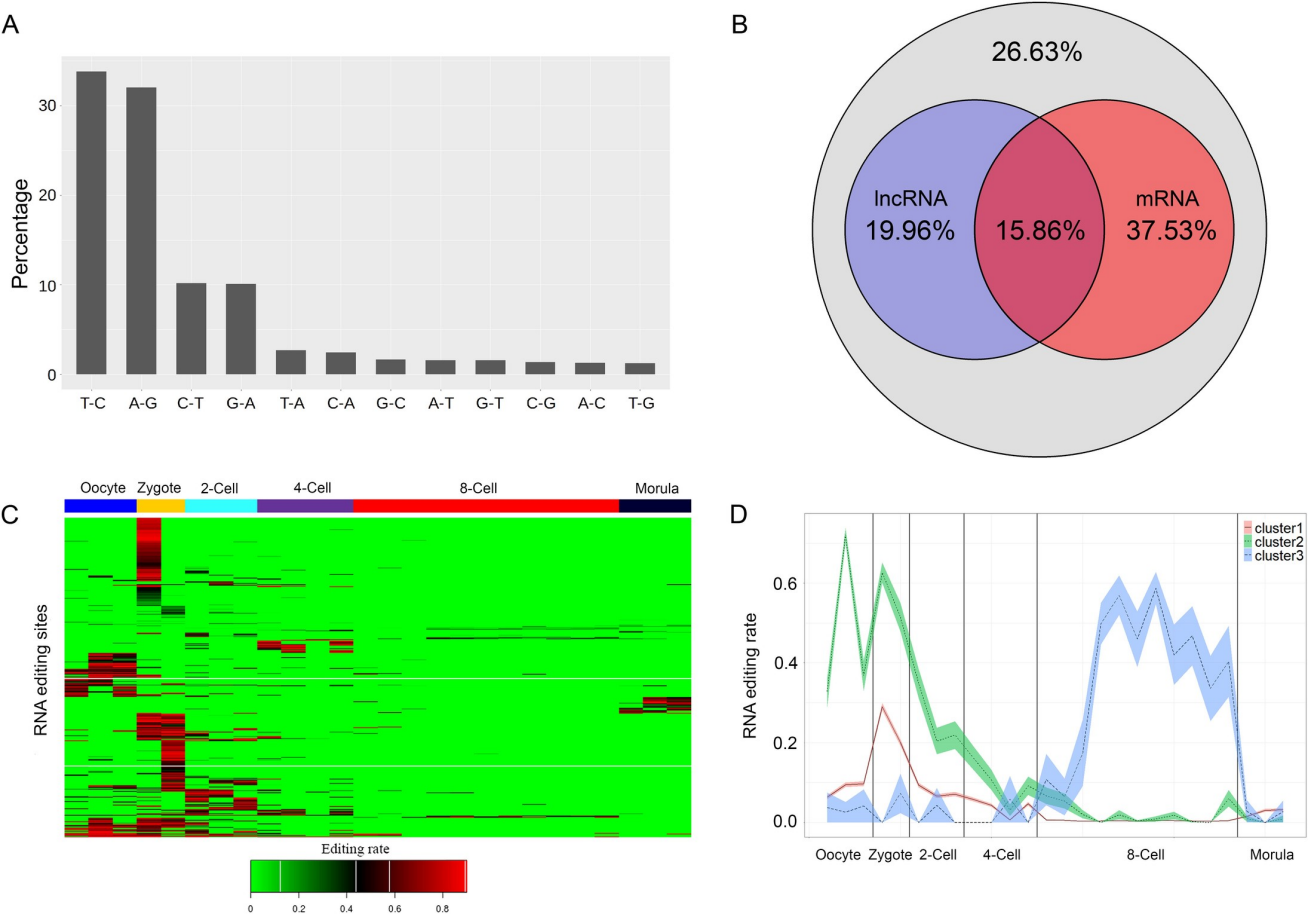
Summary of the RNA editing landscape in human early embryo development. (A) Proportions of RNA variant types in human early embryo development (B) Proportions of transcript types of RNA variants in human early embryo development. Purple means RNA editing sites having a lncRNA transcript annotation; red means RNA editing sites with an mRNA transcript annotation; and grey means RNA editing sites not annotated by lncRNA transcripts nor mRNA transcripts. (C) Heatmap of the RNA editing ratio of each differentially edited site in human early embryo development. Red means a high RNA editing ratio, while green means low or no RNA editing ratio. (D) Three clusters of differentially edited RNA editing sites. The differentially edited RNA editing sites were grouped into three clusters based on their editing pattern during human early embryo development. The Y-axis shows the mean value in each stage. The grey area shows the error bar.

A total of 5,901 RNA variant sites were identified in human early embryo development. Four major variant types were identified, including A-to-G, T-to-C, G-to-A, and C-to-T, each comprising a proportion greater than 10% (Fig 2A and S1 Table). While T-to-C and G-to-A variants were not canonical RNA editing types, they can be understood as possible A-to-I editing and C-to-U editing, respectively, if incomplete strand annotation or antisense transcription are considered [14]. On this basis, the two known RNA editing types (A-to-I and C-to-U) together accounted for the majority of RNA variants in the list (86.1%). In total, 35.82% of RNA editing sites were on lncRNA transcripts and 53.39% were on mRNAs transcripts (Fig 2B). A total of 15.86% of RNA editing sites were located on the overlap area of mRNA and lncRNA transcripts (Fig 2B). Besides, among all these RNA editing sites, A-to-G and T-to-C had around 50% non-Alu RNA editing sites and the other types were almost all non-Alu sites (S3 Fig). These results collectively indicate the successful identification of RNA editing sites using our RNA editing pipeline.

In total, 36.7% of RNA editing sites were found to have a differential editing ratio among early embryo developmental stages (Fig 2C and S1 Table). Those differentially edited sites were further grouped into 3 clusters based on the similarity of their editing pattern across different early embryo development stages, which was calculated by Lance-Williams algorithms (Fig 2D). The majority of RNA editing sites (93.2%) belonged to cluster1, followed by cluster2 (6.0%) and cluster3 (0.8%). Cluster1 and cluster2 had a similar editing pattern; specifically, they had a high RNA editing rate at the beginning of human early development, and the editing rate decreased to the bottom at the 8-cell stage (Fig 2D). Thus, most of the differentially edited RNA editing sites (99.2%) had a markedly low editing pattern at the 8-cell stage. The RNA editing sites of cluster3 were limited (0.8%) and had a different editing pattern. Cluster3 RNA editing sites had a low editing rate before the 8-cell stage and then increased to the top during the 8-cell stage development (Fig 2D). Thus, overall, the editing rate of all three clusters changed their tendency at the 8-cell stage, which indicated that the 8-cell stage is a specific period when RNA editing status changes. The RNA editing rate either increased to the maximum level or decreased to the minimum level at the 8-cell stage. These results are in agreement with previous studies that reported that the 8-cell stage is when ZGA happens [40].

### lncRNA RNA editing correlates with the expression of exons

Our study found that the majority of the differentially edited RNA editing sites were splicing sites (94.7%). Compared to non-splicing sites, differentially edited RNA editing sites were significantly over-represented on splicing sites (odds ratio = 1.5, P = 0.002; S2 Table). However, there was no difference between TFBS-related RNA editing sites and non-TFBS-related RNA editing sites (S2 Table). And the results were all confirmed in non-Alu editing sites (S2 Table).

The lncRNA RNA editing sites were more likely to be on RNA splicing sites (odds ratio = 1.97, P = 1.37×10^−8^; S3 Table). As a comparison, the mRNA RNA editing sites were less likely to be on RNA splicing sites (odds ratio = 0.12, P = 4.84×10^−62^; S3 Table). The same tendency was observed in non-Alu editing sites (S3 Table).

The association between exon expression level and RNA editing rate was analyzed. It was found that RNA editing sites located on splicing sites were more frequent on differentially expressed exons in both mRNA and lncRNA transcripts (mRNA, P = 0.0002; lncRNA, P = 0.0007; S4 Table). In mRNA, 91% of mRNA editing sites located on splicing sites occurred on differentially expressed exons, while the ratio in lncRNA was higher (it reached 97%).

**Fig 3:**
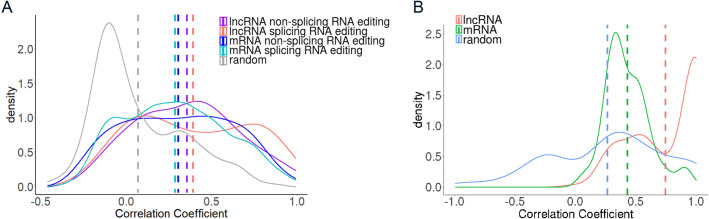
RNA editing regulates RNA splicing. (A) Distribution of the correlation coefficient between the RNA editing ratio and the expression level of each exon. The dash lines are the mean value of each type. The random is based on 10,000 random pairs of RNA editing ratio and exon expression level. (B) Distribution of correlation coefficient between RNA editing ratio and PSI of each exon. The dash lines are the mean value of each type. The random is based on 10,000 random pairs of RNA editing ratio and PSI level. PSI, percentage spliced index.

Next, the correlation coefficient between exon expression level and RNA editing rate was calculated. The correlation between exon expression level and lncRNA splicing RNA editing rate was significantly higher than that of baseline (random) (lncRNA splicing, mean R = 0.36; random, mean R = 0.06; P = 0.0001; Fig 3A). And it was also higher than that of lncRNA non-splicing RNA editing sites (lncRNA splicing, mean R = 0.36; lncRNA non-splicing, mean R = 0.33; Fig 3A). The significant correlation between exon expression level and lncRNA splicing RNA editing rate was also confirmed by the analysis which was based on only known RNA editing sites from RADAR[41] and REDIportal [42] (S4A Fig). Both the correlation of mRNA splicing RNA editing sites and mRNA non-splicing RNA editing sites were lower than that of lncRNA splicing RNA editing sites (mRNA splicing, mean R = 0.26; mRNA non-splicing, mean R = 0.28; Fig 3A).

To confirm our results, we validated our analysis in an independent dataset GSE101571. First of all, we confirmed the tendency that the lncRNA RNA editing sites were more likely to be on RNA splicing sites (odds ratio = 2.21, P = 4.02×10^−9^; S5 Table). As the comparison, the mRNA RNA editing sites were less likely to be on RNA splicing sites (odds ratio = 0.26, P = 2.54×10^−27^; S5 Table). Secondly, we confirmed RNA editing sites located on splicing sites were more frequent on differentially expressed exons in both mRNA and lncRNA transcripts (mRNA, P = 8.69×10^−10^; lncRNA, P = 0.0006; S6 Table). In mRNA, 93% of mRNA editing sites located on splicing sites occurred on differentially expressed exons, while the ratio in lncRNA was higher (it reached 97%). Thirdly, we confirmed the significantly stranger correlation between exon expression level and lncRNA splicing RNA editing rate (S5 Fig).

Combining these results, it was concluded that lncRNA RNA editing sites were more likely to be splicing sites and that RNA editing on lncRNAs can regulate the expression of the corresponding exons. Thus, it can be further assumed that RNA editing can regulate lncRNA transcript splicing.

### lncRNA RNA editing regulates lncRNA transcript splicing

To confirm our hypothesis that RNA editing can regulate lncRNA transcript splicing, the PSI of the exons, which can reflect the transcript splicing of the exons, was calculated.

The correlation coefficient between RNA editing rate and PSI on exon level was calculated, and it was found that the correlation coefficient in lncRNA was higher than that in mRNA (mRNA, mean R = 0.43; lncRNA, mean R = 0.75; P = 4.90x10^-16^) (Fig 3B). And both mRNA and lncRNA had a higher correlation coefficient than baseline (random) (random, mean R = 0.22) (Fig 3B). The result based on only known RNA editing sites from RADAR[41] and REDIportal[42] validated the high correlation between lncRNA RNA editing rate and PSI (S4B Fig). And, again, the high correlation between lncRNA RNA editing rate and PSI was confirmed by the independent dataset GSE101571 (S5 Fig).

These results indicated that RNA editing can regulate transcript splicing. The positive correlation coefficient meant that exons with a high RNA editing rate were more likely to be included in the transcript, while exons with a low RNA editing rate were more likely to be excluded, and this effect was more significant in lncRNA than in mRNA.

### Functional enrichment analysis of RNA editing-regulated lncRNAs

To study the functions of RNA editing-regulated lncRNAs, GO annotation enrichment of lncRNAs was performed. RNA editing-regulated lncRNAs were defined as those lncRNAs on which RNA editing happened on splicing sites.

**Fig 4:**
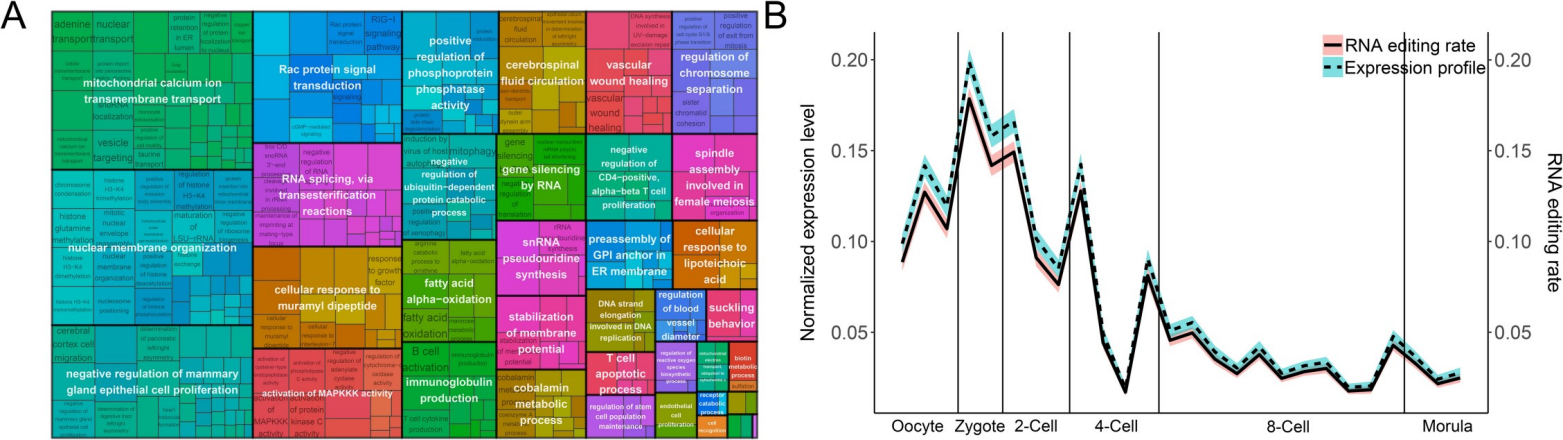
Functions enrichment analysis of RNA editing-regulated lncRNAs. (A) GO enrichment result of RNA editing-regulated lncRNAs. LncRNAs are first annotated by lncRNA2GOA [37]. Enrichment analysis was performed with the R package topGO [38]. Treemap of GO enrichment result was drawn with R package rrvgo [39]. (B) Correlation between the expression level and the RNA editing rate of RNA editing-regulated lncRNAs. The expression levels of lncRNAs were normalized by unitization with z-score normalization. The Y-axis shows the mean value in each stage, while the grey area shows the error bar.

Firstly, those lncRNAs were found enriched for the regulation of transcript expression: RNA splicing, via transesterification reactions and gene silencing by RNA (Fig 4A). The regulation of transcript expression plays important role in ZGA, during which great change in expression profile happens.

Secondly, it was found that those RNA editing-regulated lncRNAs engaged in signal transduction. For example, the activation of MAPKKK activity and Rac protein signal transduction, both of which already are important players during embryonic development (Fig 4A) [43].

Thirdly, those RNA editing regulated lncRNAs were found co-expressed with the mRNAs which have a function in the transmembrane transport of mitochondrial calcium ion (Fig 4A). The previous study already found that calcium ion uptake and release was an important factor in the regulation of oocyte and embryo development [44]. Increaseing in calcium ion was known to take place at different stages of development, including during cleavage to the two-cell stage [44,45].

Next, our study evaluated the expression levels of those RNA editing-regulated lncRNAs and their RNA editing rates. Overall, the expression profile and RNA editing rates of those lncRNAs shared a similar but special pattern. Both had a high value/ratio at the beginning of embryo development, which decreased to the minimum after the 4-cell stage. A significant correlation between RNA editing rate and expression profile was observed (P < 2.2x10^-16^) (Fig 4B).

All these results indicated that RNA editing can affect distinct key events during human early embryo development by regulating relative lncRNAs.

## Discussion

Previous studies found there was a great change in gene expression profile during human embryo development. LncRNAs were found to play roles in mammal early embryo development, including human and mouse [2]. LncRNAs have a stage-specific expression pattern during early embryo development [5], which is related to human oocyte maturation and human ZGA [5]. Meanwhile, RNA editing was found to be able to affect the expression of lncRNAs [10]. However, the detailed mechanism behind the regulation of RNA editing on lncRNAs remained unclear.

In our study, through analyzing single cell data, it was found that RNA editing could affect the transcript splicing of lncRNAs during human early embryo development. Firstly, it was found that, during human early embryo development, the differentially edited RNA editing sites were over-represented on splicing sites. LncRNA editing sites were found more likely to be on the RNA splicing sites, while the mRNA editing sites were found less likely to be on the RNA splicing sites. Furthermore, the RNA editing rate of lncRNA exons had a significantly higher correlation coefficient with the expression level and PSI index of the lncRNA exon. It means that highly edited exons were more likely to remain in the final transcripts while lowly edited exons were not. Thus, regulation of RNA splicing might be the mechanism by which RNA editing affects lncRNA expression in human early embryo development.

The differentially edited RNA editing sites in human early embryo development could be grouped into 3 clusters. The majority (cluster1 and cluster2) shared a special pattern: Both decease to minimum levels at the 8-cell stage. Overall, all three clusters of RNA editing sites had great changes in editing rate at the 8-cell stage. They either increased to the highest level or decreased to the lowest level at the 8-cell stage, which indicated that the 8-cell stage is an important stage for human early embryo development, during which ZGA happens. During ZGA, maternal transcripts are degraded and zygotic transcription begins, which is coordinated with other embryonic events, including changes in cell cycle, chromatin state, and nuclear-to-cytoplasmic component ratios [40]. A previous study found that lncRNA transcripts have a great change in expression profile at the 8-cell stage [5]. As RNA editing can regulate transcript splicing, the differential RNA editing ratio in the 8-cell stage could be the reason for the change in lncRNA transcript expression.

The decreased editing ratio in the 8-cell stage observed in our study is in agreement with a previous study that reported that RNA editing was significantly lower in embryonic tissue than in adult tissue. Meanwhile, A-to-I editing levels in various human cancer types revealed general hypo-editing in cancer tissues [46]. A possible reason behind this could be that high RNA editing would increase the expression of detrimental transcripts such as cancer activators. The RNA editing of these transcripts remains markedly low in fetal stages, and it increases when people growing up which causes disease.

Our study also found that RNA editing may participate in numerous functions during human early embryo development by regulating the splicing of related lncRNAs. First, our study found that RNA editing can regulate lncRNAs that are related to the regulation of transcript expression including RNA splicing, via transesterification reactions and gene silencing by RNA. As we all know that there is a great change of expression profile happening at the 8-cell stage during ZGA [5], our results suggested that the RNA editing regulated lncRNAs may involve in the expression profile changing process. For example, there is a degradation of maternal transcripts in ZGA, during which lncRNAs can play a role through the function of gene silencing by RNA.

RNA editing can also affect signal transduction through lncRNAs that participate in the activation of MAPKKK (MAP3K) activity and Rac protein signal transduction. Both of them are related to MAPK pathways [47]: 1) MAPKKK is an up-stream protein of MAPK pathway; 2) Rac protein can activate an upstream MAP kinase kinase kinase kinase (MAP4K), following which the MAP kinase cascades proceed through the sequential phosphorylation of constituent MAP3K, MAP2K and MAPK [47]. MAPK pathways transmit signals from ligand-receptor interactions and convert them into a variety of cellular responses, ranging from apoptosis to embryonic development [43]. MAPK/ERK2 is not expressed in unfertilized eggs, but its expression level gradually increases from the 2-cell stage throughout the whole preimplantation embryo development [48], which corresponds to ZGA [43].

Previous studies also found that RNA editing could affect signaling pathways, which partly supported our results [49,50]. RNA editing of the GLI1 transcription factor could modulate the output of Hedgehog signaling. Adenosine to inosine substitution led to a change from arginine to glycine at position 701 that influences not only GLI1 transcriptional activity but also GLI1-dependent cellular proliferation [49]. A previous study on hepatocellular carcinoma found that RNA editing may be associated with apoptosis signaling pathways [50]. These previous results supported our finding that RNA editing regulates signaling pathways during human early embryo development.

In summary, our study found that RNA editing can regulate lncRNA splicing during development and play further roles in human early embryo development. Since RNA editing and lncRNAs are both important in multiple diseases and development processes, understanding the association between RNA editing and lncRNAs may provide a new insight into human developmental biology and common birth defects, as well as potential benefits for reproductive health and improvements in medicine.

## Supporting information

S1 Fig Comparison between different DE analysis methods. (A) DEsingle vs DEseq. The y-axis shows the overlapping ratio between the outputs of the two methods. (B) MAST vs DEseq. (C) MAST vs DEsingle.
(TIF)

S2 Fig Comparison between different RNA editing sites identification methods. X-axis means the different categories of RNA editing sites, and the y-axis is the FDR rate.
(TIF)

S3 Fig Proportions of non-Alu RNA editing sites in human early embryo development. (TIF)

S4 Fig RNA editing regulates RNA splicing (based on only known RNA editing sites from RADAR and REDIportal). (A) Distribution of the correlation coefficient between the RNA editing ratio and the expression level of each exon. The dash lines are the mean value of each type. The random is based on 10,000 random pairs of RNA editing ratio and exon expression level. (B) Distribution of correlation coefficient between RNA editing ratio and PSI of each exon. The dash lines are the mean value of each type. The random is based on 10,000 random pairs of RNA editing ratio and PSI level. PSI, percentage spliced index.
(TIF)

S5 Fig RNA editing regulates RNA splicing (based on independent dataset GSE101571). (A) Distribution of the correlation coefficient between the RNA editing ratio and the expression level of each exon. The dash lines are the mean value of each type. The random is based on 10,000 random pairs of RNA editing ratio and exon expression level. (B) Distribution of correlation coefficient between RNA editing ratio and PSI of each exon. The dash lines are the mean value of each type. The random is based on 10,000 random pairs of RNA editing ratio and PSI level. PSI, percentage spliced index.
(TIF)

S1 Table RNA editing sites have a differential editing ratio among early embryo developmental stages. (XLSX)

S2 Table Chi-square test for differential editing RNA editing sites. (DOCX)

S3 Table Chi-square test for splicing related RNA editing sites. (DOCX)

S4 Table Fisher exact test for RNA editing sites on differential expressed exon. (DOCX)

S5 Table Chi-square test for splicing related RNA editing sites(GSE101571). (DOCX)

S6 Table Fisher exact test for RNA editing sites on differential expressed exon. (DOCX)
