# Supplementary figures and images for: RNA editing regulates lncRNA splicing in human early embryo development
Source: PLoS Comput Biol. 2021 Dec 1;17(12):e1009630. doi: 10.1371/journal.pcbi.1009630 (PMC8668112; doi:10.1371/journal.pcbi.1009630)

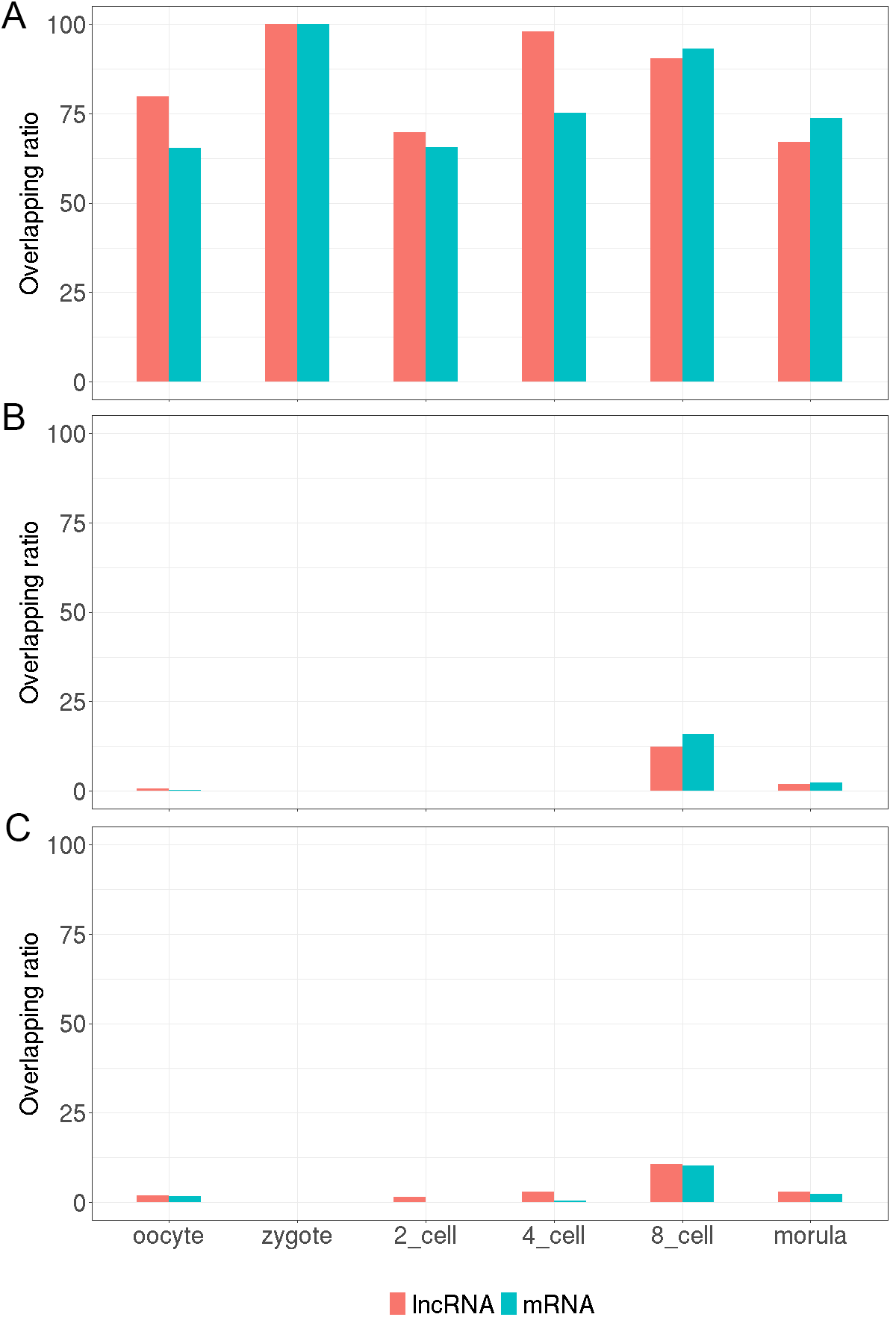

Supplement: S1 Fig — (A) DEsingle vs DEseq. The y-axis shows the overlapping ratio between the outputs of the two methods. (B) MAST vs DEseq. (C) MAST vs DEsingle. (TIF) [file pcbi.1009630.s001.tif]

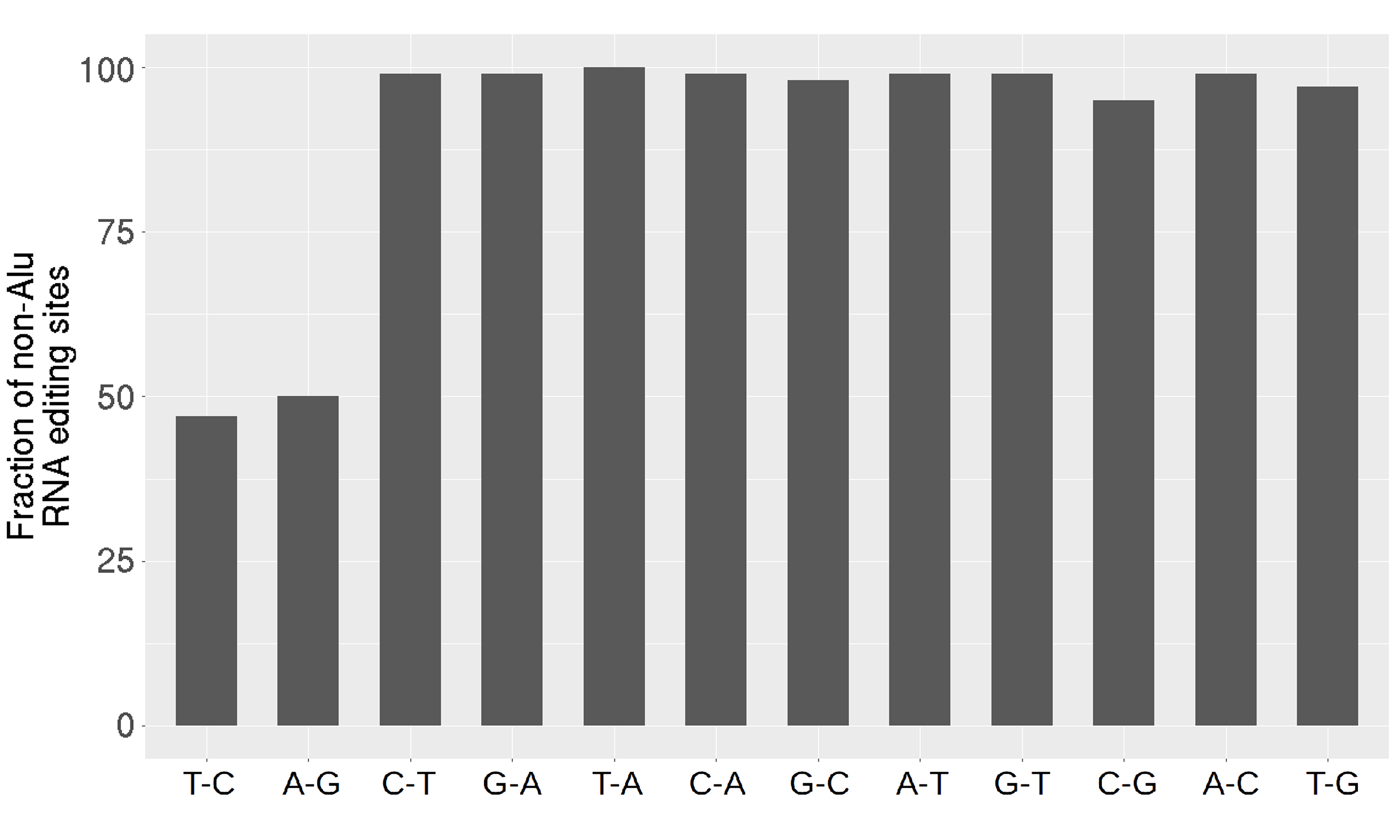

Supplement: S3 Fig — (TIF) [file pcbi.1009630.s003.tif]

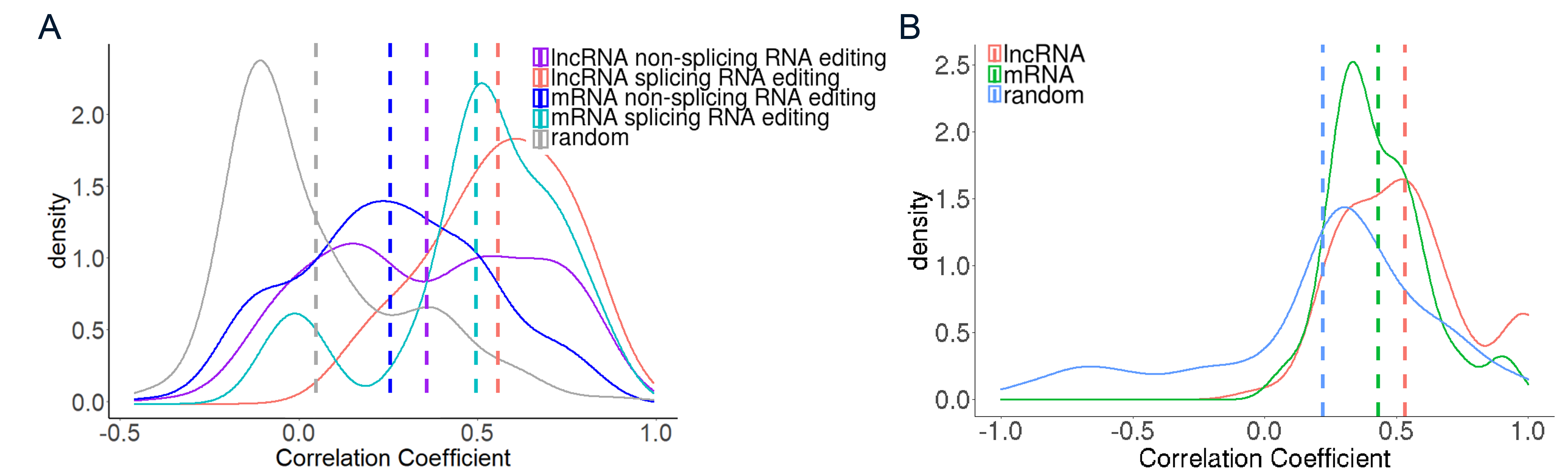

Supplement: S4 Fig — (A) Distribution of the correlation coefficient between the RNA editing ratio and the expression level of each exon. The dash lines are the mean value of each type. The random is based on 10,000 random pairs of RNA editing ratio and exon expression level. (B) Distribution of correlation coefficient between RNA editing ratio and PSI of each exon. The dash lines are the mean value of each type. The random is based on 10,000 random pairs of RNA editing ratio and PSI level. PSI, percentage spliced index. (TIF) [file pcbi.1009630.s004.tif]

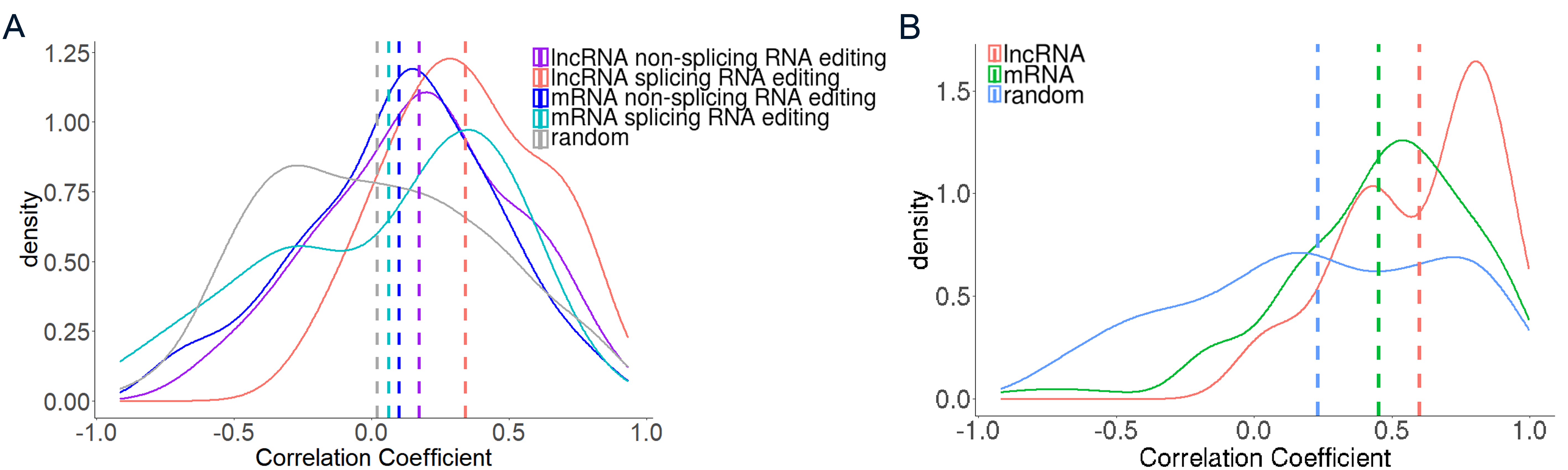

Supplement: S5 Fig — (A) Distribution of the correlation coefficient between the RNA editing ratio and the expression level of each exon. The dash lines are the mean value of each type. The random is based on 10,000 random pairs of RNA editing ratio and exon expression level. (B) Distribution of correlation coefficient between RNA editing ratio and PSI of each exon. The dash lines are the mean value of each type. The random is based on 10,000 random pairs of RNA editing ratio and PSI level. PSI, percentage spliced index. (TIF) [file pcbi.1009630.s005.tif]
