# Supplementary material for: RNA editing regulates lncRNA splicing in human early embryo development
Source: PLoS Comput Biol. 2021 Dec 1;17(12):e1009630. doi: 10.1371/journal.pcbi.1009630 (PMC8668112; doi:10.1371/journal.pcbi.1009630)
Supplement: S2 Table — (DOCX) [file pcbi.1009630.s007.docx]

**Table S2 Chi-square test for differential editing RNA editing sites**

| Type of RNA editing sites |  |  | Differential edited RNA editing sites | Non-differential edited RNA editing sites | P-value | Odd Ratio |
| --- | --- | --- | --- | --- | --- | --- |
| All the RNA editing sites | Splicing sites | Splicing RNA editing sites | 1501 | 3984 | 0.002 | 1.49 |
|  |  | Non-splicing RNA editing sites | 84 | 332 |  |  |
|  | TFBS | TFBS RNA editing sites | 53 | 158 | 0.62 | 0.91 |
|  |  | Non-TFBS RNA editing sites | 1532 | 4158 |  |  |
| Non-Alu RNA editing sites | Splicing sites | Splicing RNA sites | 845 | 2648 | 0.02 | 1.37 |
|  |  | Non-splicing RNA editing sites | 74 | 317 |  |  |
|  | TFBS | TFBS RNA editing sites | 53 | 158 | 0.67 | 1.08 |
|  |  | Non-TFBS RNA editing sites | 866 | 2807 |  |  |
