# Supplementary material for: RNA editing regulates lncRNA splicing in human early embryo development
Source: PLoS Comput Biol. 2021 Dec 1;17(12):e1009630. doi: 10.1371/journal.pcbi.1009630 (PMC8668112; doi:10.1371/journal.pcbi.1009630)
Supplement: S3 Table — (DOCX) [file pcbi.1009630.s008.docx]

**Table S3 Chi-square test for splicing related RNA editing sites**

| Type of RNA eiditng sites |  |  | Splicing related RNA editing sites | Non-splicing related RNA editing sites | P-value | Odd Ratio |
| --- | --- | --- | --- | --- | --- | --- |
| All the RNA editing sites | lncRNA | lncRNA RNA editing sites | 2019 | 95 | 1.37x10^-8^ | 1.97 |
|  |  | Non lncRNA RNA editing sites | 3466 | 321 |  |  |
|  | mRNA | mRNA RNA editing sites | 2777 | 374 | 4.84x10^-62^ | 0.12 |
|  |  | Non mRNA RNA editing sites | 2708 | 42 |  |  |
| Non-Alu RNA editing sites | lncRNA | lncRNA RNA editing sites | 1353 | 90 | 3.55x10^-10^ | 2.11 |
|  |  | Non lncRNA RNA editing sites | 2140 | 301 |  |  |
|  | mRNA | mRNA RNA editing sites | 1783 | 360 | 1.25x10^-63^ | 0.09 |
|  |  | Non mRNA RNA editing sites | 1710 | 31 |  |  |
