# Supplementary material for: RNA editing regulates lncRNA splicing in human early embryo development
Source: PLoS Comput Biol. 2021 Dec 1;17(12):e1009630. doi: 10.1371/journal.pcbi.1009630 (PMC8668112; doi:10.1371/journal.pcbi.1009630)
Supplement: S4 Table — (DOCX) [file pcbi.1009630.s009.docx]

**Table S4 Fisher exact test for RNA editing sites on differential expressed exon**

| Type of RNA eiditng sites |  |  | RNA editing sites on differential expressed exon | RNA editing sites on non-differential expressed exon | P-value | Odd Ratio |
| --- | --- | --- | --- | --- | --- | --- |
| All the RNA editing sites | lncRNA | lncRNA splicing related RNA editing sites | 953 | 1066 | 0.0007 | 2.14 |
|  |  | Non-lncRNA splicing related RNA RNA editing sites | 28 | 67 |  |  |
|  | mRNA | mRNA splicing related RNA RNA editing sites | 952 | 1825 | 0.0002 | 1.60 |
|  |  | Non-mRNA splicing related RNA RNA editing sites | 92 | 282 |  |  |
| Non-Alu RNA editing sites | lncRNA | lncRNA splicing related RNA editing sites | 634 | 719 | 0.0004 | 2.29 |
|  |  | Non-lncRNA splicing related RNA RNA editing sites | 25 | 65 |  |  |
|  | mRNA | mRNA splicing related RNA RNA editing sites | 612 | 1171 | 4.41x10^-5^ | 1.71 |
|  |  | Non-mRNA splicing related RNA RNA editing sites | 84 | 276 |  |  |
