# Supplementary material for: RNA editing regulates lncRNA splicing in human early embryo development
Source: PLoS Comput Biol. 2021 Dec 1;17(12):e1009630. doi: 10.1371/journal.pcbi.1009630 (PMC8668112; doi:10.1371/journal.pcbi.1009630)
Supplement: S5 Table — (DOCX) [file pcbi.1009630.s010.docx]

**Table S5 Chi-square test for splicing related RNA editing sites(GSE101571)**

| Type of RNA eiditng sites |  |  | Splicing related RNA editing sites | Non-splicing related RNA editing sites | P-value | Odd Ratio |
| --- | --- | --- | --- | --- | --- | --- |
| All the RNA editing sites | lncRNA | lncRNA RNA editing sites | 1313 | 74 | 4.02x10^-9^ | 2.21 |
|  |  | Non lncRNA RNA editing sites | 2133 | 263 |  |  |
|  | mRNA | mRNA RNA editing sites | 1606 | 260 | 2.54x10^-27^ | 0.26 |
|  |  | Non mRNA RNA editing sites | 1820 | 77 |  |  |
| Non-Alu RNA editing sites | lncRNA | lncRNA RNA editing sites | 1180 | 73 | 2.90x10^-10^ | 2.28 |
|  |  | Non lncRNA RNA editing sites | 1861 | 262 |  |  |
|  | mRNA | mRNA RNA editing sites | 1414 | 258 | 3.47x10^-27^ | 0.26 |
|  |  | Non mRNA RNA editing sites | 1627 | 77 |  |  |
