# Supplementary material for: RNA editing regulates lncRNA splicing in human early embryo development
Source: PLoS Comput Biol. 2021 Dec 1;17(12):e1009630. doi: 10.1371/journal.pcbi.1009630 (PMC8668112; doi:10.1371/journal.pcbi.1009630)
Supplement: S6 Table — (DOCX) [file pcbi.1009630.s011.docx]

**Table S6 Fisher exact test for RNA editing sites on differential expressed exon**

| Type of RNA eiditng sites |  |  | RNA editing sites on differential expressed exon | RNA editing sites on non-differential expressed exon | P-value | Odd Ratio |
| --- | --- | --- | --- | --- | --- | --- |
| All the RNA editing sites | lncRNA | lncRNA splicing related RNA editing sites | 542 | 771 | 0.0006 | 2.14 |
|  |  | Non-lncRNA splicing related RNA RNA editing sites | 16 | 58 |  |  |
|  | mRNA | mRNA splicing related RNA RNA editing sites | 499 | 1107 | 8.69x10^-10^ | 2.90 |
|  |  | Non-mRNA splicing related RNA RNA editing sites | 35 | 225 |  |  |
| Non-Alu RNA editing sites | lncRNA | lncRNA splicing related RNA editing sites | 459 | 721 | 0.0004 | 2.27 |
|  |  | Non-lncRNA splicing related RNA RNA editing sites | 16 | 57 |  |  |
|  | mRNA | mRNA splicing related RNA RNA editing sites | 422 | 992 | 1.45x10^-8^ | 2.71 |
|  |  | Non-mRNA splicing related RNA RNA editing sites | 35 | 223 |  |  |
